# A Curious Disease Called “Head-Drop”

**Published:** 1888-02

**Authors:** K. Nakano


### A CURIOUS DISEASE, CALLED "HEAD-DROP."

BY DR. K. NAKANO.

In the region where I live there is a curious disease, called "head-drop," of which I heard many years since. At first, I supposed it to be a symptom of some disease, and so I did not bear it in mind. But the more I heard of the strangeness of the case, the more I desired to examine into it.

Last year, when cholera was prevailing, I was going the round of the villages for inspection, when I came to a house, the inmates of which were all in bed. Upon inquiry I discovered that they were affected with this "head-drop." As it had been my long desire, I immediately examined them. The case is, indeed, curious, but there is no other particular symptom to point out. I gave 5 grams of sulphate of quinine, and I was told that they were cured, two weeks after. I therefore pronounced the disease to be curable, and so many patients came to me that at last I hung up a notice regarding the treatment of head-drop, in January of this year.

*Name and history.*—Head-drop is the name by which the disease is called among the native people, and its history is not known. But from ancient time there were great numbers of patients, and, when attacked by the disease, the people, believing it to be incurable, did not take any pains to consult a physician; hence, nobody has investigated its pathology, and, accordingly, it has no proper name. I think the above is a name given from its outward appearance, and that it is a kind of endemic disease.

*Character of the region of occurrence.*—The region where this disease occurs is in wet places between mountains, where the current of air is not pure and the sun does not shine all the time; hence, there are no cases found by the sea shore or in high, dry regions. Moreover, those who live in such places are farmers, who generally eat bad food and drink bad water. So far as my experience goes, it seems there is no connection as to sex or natural constitution.

*Condition of prevalence and cause of the disease.*—It occurs in all seasons, but especially from the end of spring to the beginning of summer. When it prevails it does not attack all the people of the region, and even if in one village one family is affected, there may be none in neighboring families, and the family which was formerly affected by the disease often gets rid of it, and another in turn becomes the diseased family; hence, it seems to be not hereditary. Most people do not like to have communication with such a family. Again, if one person in a family is attacked by the disease, the rest of those in the house are usually affected also, but there is no evidence of affection by contact.

As to the cause of the disease I do not yet know, on account of my short experience. Judging from the symptoms of the attacks, I think there is no doubt that some kind of miasma or malaria attacks a part of the brain or spinal cord. It is not plain whether it causes anæmia or congestion, or attacks by exuding serous pigment, but there must be some part where congestion is caused. In mild cases it attacks a part of the medulla oblongata and corpora quadrigemina of brain, and in severe cases it attacks the end of the spinal cord severely by passing through it. It is said that one who is hungry is more easily affected. In the district around, there are a great many cases of ague every year, but it is curious that there are almost none in the place where this disease occurs, although the circumstances favor the occurrence of ague hence, from the first, I thought it to be malarious cerebral disease.

*Symptoms.*—There are no prognostic signs, except uneasiness of mind and headache a few hours before the occurrence; but it comes suddenly, and the head becomes so heavy that it can not be kept upright, and so hangs down in front, and walking becomes unsteady, too. In some cases the congestion of conjunctiva and dilatation of pupils(?) produce double sight in one eye, and feeling in the neck becomes somewhat dull. In severe cases the patient has double sight in both eyes, and cannot walk in the sunshine. Moreover, the tongue becomes stiff, the speech slow, swallowing difficult, the loins paralyzed, and walking becomes difficult. The specific gravity of the urine discharged is somewhat heavy, the color transparent white, and white sediment appears after standing for a few hours. If it is warmed, or some acid is put in, it neither coagulates nor dissolves. If we examine it with the microscope, we observe two or three pieces of epithelium of the mucous membrane of the bladder, beside cloudy substances, while there is no trace of urea. If we examine for sugar by Trommer's test and subnitrate of bismuth, we often get reaction. Again, the case becomes so severe that at last one of the legs is paralyzed and loses sensation.

The following is the comparative table of patients:

| | | | |
|---|---|---|---|
| 63 | Men—37 | Below age of 15 | —12 |
| | | " " 25 | —29 |
| | | " " 35 | —14 |
| | Women—26 | " " 45 | — 5 |
| | | " " 60 | — 2 |

Note.—These 63 were all farmers, among whom were two men who were affected after being attacked by masked ague, but there have been no deaths from this disease alone.

*Duration and prognosis.*—Most are attacked when hungry, and continue to be affected from a few hours to a few weeks, and sometimes to two or three months. If any one is attacked once, he is apt to be attacked again, even in the same year. So the prognosis is said to be rather bad, though there is no pain to be felt. During the interval, the health of the patient does not differ from usual, and in mild cases little work is performed generally.

*Treatment.*—The patient feels no pain. The native people, anticipating its duration, do not consult a physician; hence I have not heard of any method of treatment. Before or during the occurrence, I gave pills of quinine and iron, with tonic medicines, and tried to check the progress of the disease by using quinine. Again, I tried stimulant on diseased parts, especially subcutaneous injection of camphor, and also irritative medicines, such as injection of strychnine, internal and external treatment of iodine and mercury, or applied counter-irritants, leeches, cupping glasses, blisters, and electricity; but some were cured and some not. And even those who were cured, it is perhaps only a temporary relief. It is curious to say that one who is affected when he is hungry, is cured immediately by taking much food.—*The Sei-I-Kwai Medical Journal, Tokyo, Japan.*

